# Effect of Allopurinol Use on Kidney Function Among Patients with Gout and Chronic Kidney Disease

**DOI:** 10.3390/gucdd3030013

**Published:** 2025-07-10

**Authors:** Ana Beatriz Vargas-Santos, Christine E. Peloquin, Tuhina Neogi

**Affiliations:** 1 Rheumatology Unit, Hospital Universitário Pedro Ernesto, Universidade do Estado do Rio de Janeiro, Rio de Janeiro 20551-030, RJ, Brazil; 2 Section of Rheumatology, Boston University School of Medicine, Boston, MA 02118, USA

**Keywords:** gout, chronic kidney disease, renal function, allopurinol, hyperuricemia

## Abstract

The evidence regarding allopurinol’s effects on renal function among people with hyperuricemia and gout has been conflicting, though clinicians are often cautious about using allopurinol in chronic kidney disease (CKD). We sought to examine the relation between allopurinol use in those with gout and CKD and the risk of worsening renal function. We conducted a time-stratified propensity score (PS)-matched cohort study on the IQVIA Medical Research Data representative of the UK general population. Among participants 18–89 years old with gout and CKD 3–4 not on urate-lowering therapy within one year prior, we identified new users of allopurinol and matched them 1:1 with a non-user. We analyzed the relation between incident allopurinol use and the changes in the eGFR at one year of follow-up using linear regression adjusted for the potential confounders included in the PS model. We PS-matched 10,716 allopurinol initiators to 10,716 non-users, among whom 42% were female, the mean age was 74 years and 7% had CKD4. The progression to dialysis or kidney transplant was similar in both groups. The mean eGFR prior to the study entry was 48.4 mL/min among allopurinol initiators and 49.5 mL/min among non-users, while the last eGFR within one year was 49.4 and 49.7 mL/min, respectively. The allopurinol initiators had an adjusted mean increase in the eGFR of 0.81 mL/min (95% CI 0.57–1.05) greater than that of non-users. Among those with gout and CKD 3–4, allopurinol did not worsen renal function and may have slightly improved it, suggesting that allopurinol is not detrimental to patients with gout who have CKD.

## 1. Introduction

There is an ongoing debate regarding whether treating hyperuricemia may be renoprotective [1,2]. Hyperuricemia may be harmful to the kidneys through different mechanisms: activation of the renin-angiotensin-aldosterone system, activation of other vasoconstrictors (endothelin and thromboxane), suppression of vasodilators (nitric oxide), oxidative stress, production of inflammatory cytokines, and proliferation of vascular smooth muscle cells, resulting in glomerular and possible tubular damage [1,3]. Gout itself, apart from hyperuricemia, may additionally contribute to impairing renal function through inflammatory flares, frequent non-steroidal anti-inflammatory drugs (NSAID) use, and urate crystals deposition in renal and vascular tissues [4]. Effectively treating hyperuricemia in the context of gout could thus reduce renal damage.

However, gout is commonly underdiagnosed and undertreated. For instance, studies show that urate-lowering therapy (ULT) is prescribed to only around 33% of gout patients in the United States and approximately 38% of prevalent gout patients in the United Kingdom, highlighting a significant gap in optimal care [5,6]. The presence of CKD further compounds the inadequate management of gout due to factors such as the reduced efficacy of uricosuric drugs in this condition, and the longstanding practice of renal-dosing allopurinol according to creatinine clearance, when it is well-demonstrated that this dose reduction results in only around 40% of patients achieving the serum urate (SU) target of <6 mg/dL without meaningfully diminishing the risk of hypersensitivity [7–9]. On the other hand, there are data to suggest that ULT may have renoprotective benefits [1,10].

Recently, the G-CAN (Gout, Hyperuricemia and Crystal-Associated Disease Network) published systematic reviews on the management of gout in CKD [11,12] and a consensus statement on the research priorities for this topic [13]. One of the three general research priorities is the focus of the present study: “Does the treatment of gout and treat-to-target management of gout reduce progression of CKD and/or improve renal function?”. This study aimed to evaluate the relation between incident allopurinol use and changes in the estimated glomerular filtration rate (eGFR) among participants with gout and CKD stage 3–4 at the start of therapy.

## 2. Materials and Methods

### 2.1. Study Design and Setting

We conducted a propensity-score matched cohort study on the IQVIA Medical Research Data (IMRD). IMRD contains longitudinal non-identified patient electronic healthcare records (EHR) collected from UK general practitioner (GP) clinical systems incorporating data from THIN, a Cegedim database. IMRD includes data from over 17 million patient records. The database holds patient demographic information, prescribed medication, signs, diagnoses, lab tests and additional information such as lifestyle factors, BMI and vaccinations as recorded in GP practices.

The use of IMRD for research has been approved by the NHS Health Research Authority (NHS Research Ethics Committee ref 23/EM/0151) for medical and public health research. This study was approved by the IQVIA Medical Research Data Review Committee (SRC approval reference 12-005) and the Institutional Review Board at Boston University Medical Center.

### 2.2. Participants

We selected participants aged 18 to <90 years, diagnosed with gout and CKD stage 3 or 4, who had been enrolled with their GP for at least one year prior to the study entry from 1 January 2000 to 31 December 2018. The study entry date was the date when a participant met all these criteria.

The diagnosis of gout was based on a gout Read code. Participants were required to have at least one eGFR value within two years prior to the follow-up start date and at least one eGFR during the first year of follow-up. We excluded participants with CKD stage 5 or on renal replacement therapy (peritoneal dialysis, hemodialysis, or renal transplant) prior to the study entry date. CKD stage 5 was defined as a CKD5 Read code or eGFR < 15 mL/min on at least two occasions more than 90 days apart within one year with no intervening eGFR ≥ 20 mL/min. Then, CKD stage 3 or 4 was defined as a Read code for CKD stage 3 or 4, or as eGFR < 60 mL/min on at least two occasions more than 90 days apart within one year with no intervening eGFR ≥ 75 mL/min; for the latter definition, the date of diagnosis was the date of the second qualifying eGFR. Participants with ULT use (allopurinol, febuxostat, probenecid, or sulfinpyrazone) within the year prior to study entry were also excluded. From this sample, incident allopurinol users were identified.

**Figure 1.**
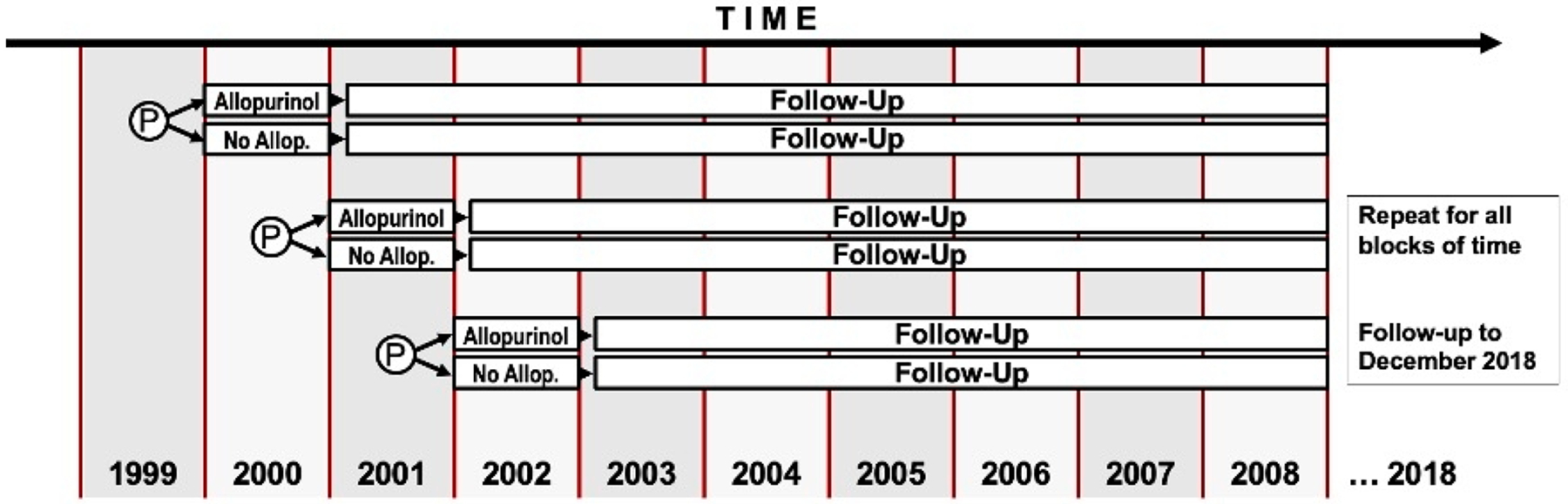
Study design: 1-year cohort accrual blocks with 1:1 propensity-score greedy matching. P: matched pair.

To account for secular trends, we created one-year cohort accrual blocks (Figure 1). The index date was defined as the date of the first allopurinol prescription within the study time for allopurinol initiators and a randomly assigned date within the one-year accrual block for comparators. We computed propensity scores to minimize the effects of confounding by indication using logistic regression, in which allopurinol initiation was the dependent variable and the independent variables were potential confounders that reflect indication for allopurinol use and/or risk of CKD progression. Each allopurinol initiator was greedy matched to an allopurinol non-user in a ratio of one to one using the propensity score within 1-year cohort accrual blocks.

Before the propensity score matching process, we excluded participants with the following conditions at any time prior to the index date: (1) other organ or bone marrow transplant (Read codes); (2) primary kidney disease (including polycystic kidney disease) or systemic vasculitis that affects the kidneys (Read codes); (3) cirrhosis (Read codes); (4) multiple myeloma or renal carcinoma (Read codes); and (5) CKD stage 5 (Read codes or the eGFR definition mentioned above). We also excluded participants with the following criteria within one year prior to the index date: (1) use of ULT other than allopurinol; (2) active cancer other than “in situ” cancers, squamous skin cancer, or basal skin cancer; and (3) no contact with the health care system (i.e., no appointment with the GP, no laboratory test, and no prescription). Additionally, we excluded participants with no data on body mass index (BMI) at any time before the index date. For those with no SU value available within the period of 5 years prior to the index date, we used the first SU value within 30 days after the index date; if SU was still unavailable, the participant was then excluded.

### 2.3. Variables

The exposure was incident allopurinol use, defined as allopurinol initiation with no allopurinol use within one year prior to study entry. Comparators were participants with no allopurinol use prior to or during the 1-year accrual block.

The Covariates included in the propensity score were (detailed in Table S1): (1) age at the index date; (2) gender; (3) most recent BMI prior to the index date; (4) baseline CKD stage 4 based on Read codes or—if no Read code available—based on the eGFR; (5) diagnosis of cardiovascular disease or heart failure or hypertension based on Read codes; (6) diabetes mellitus defined as diagnostic Read code or diabetes drug prescription; (7) medication use based on prescription data (diuretics (loop, thiazides or thiazide-like), angiotensin-converting-enzyme inhibitors/non-losartan angiotensin II receptor blockers, losartan, colchicine, NSAIDs, low-dose aspirin for cardiovascular disease prevention); (8) number of visits to the GP within one year prior to the index date; (9) hospitalization within one year prior to the index date; and (10) most recent SU within 5 years prior to the index date (if none were available, we used the first SU value within 30 days after the index date). Read codes for comorbidities were considered at any time prior to the index date, and prescription data were assessed within one year prior to the index date.

The **outcome** was the mean difference in the eGFR change within one year. For each group, the change in eGFR was calculated as the post-index eGFR (most recent eGFR within 1-year post-index date and prior to the study end date) minus the pre-index eGFR (the most recent eGFR within two years prior to or on the index date). We used the Modification of Diet in Renal Disease (MDRD) formula to calculate the eGFR [14]. The analysis of the serum creatinine values for computation of the eGFR and the use of Read codes for CKD in the IQVIA Medical Research Data have been previously validated [15].

### 2.4. Statistical Analysis

We aimed to assess the mean change in the eGFR at one year after allopurinol initiation in an intention-to-treat analytic approach. The follow-up time started at the index date and continued until the last eGFR within one year. For those who underwent dialysis or a renal transplant before one year of follow-up was complete, we used the last eGFR before dialysis or renal transplant. The follow-up was also terminated at the time of death, transfer out of the GP practice, date of last data collection by the GP, when a participant turned 90 years old, or end of the study (31 December 2018), with the last eGFR prior to these occurrences used in the analyses.

We analyzed the relation between incident allopurinol use and the changes in the eGFR using a normal linear regression model (SAS procedure PROC GENMOD, normal distribution) with an intention-to-treat approach. We additionally adjusted for the covariates included in the propensity score. The allopurinol dose prescribed was evaluated among the initiators. Because the participants could stop using allopurinol and non-users could start using the medication, we performed a sensitivity analysis censoring participants at an exposure status change. The assessment of covariate balance was performed (Methods Supplement, Table S2). To help substantiate the anticipated questions related to potential outcomes, some post-baseline characteristics were described for both groups (Table S3).

All analyses were performed using SAS 9.4 (SAS Institute, Cary, NC, USA). The *p*-values were two-sided and considered significant if <0.05.

## 3. Results

### 3.1. Cohort Selection

**Figure 2.**
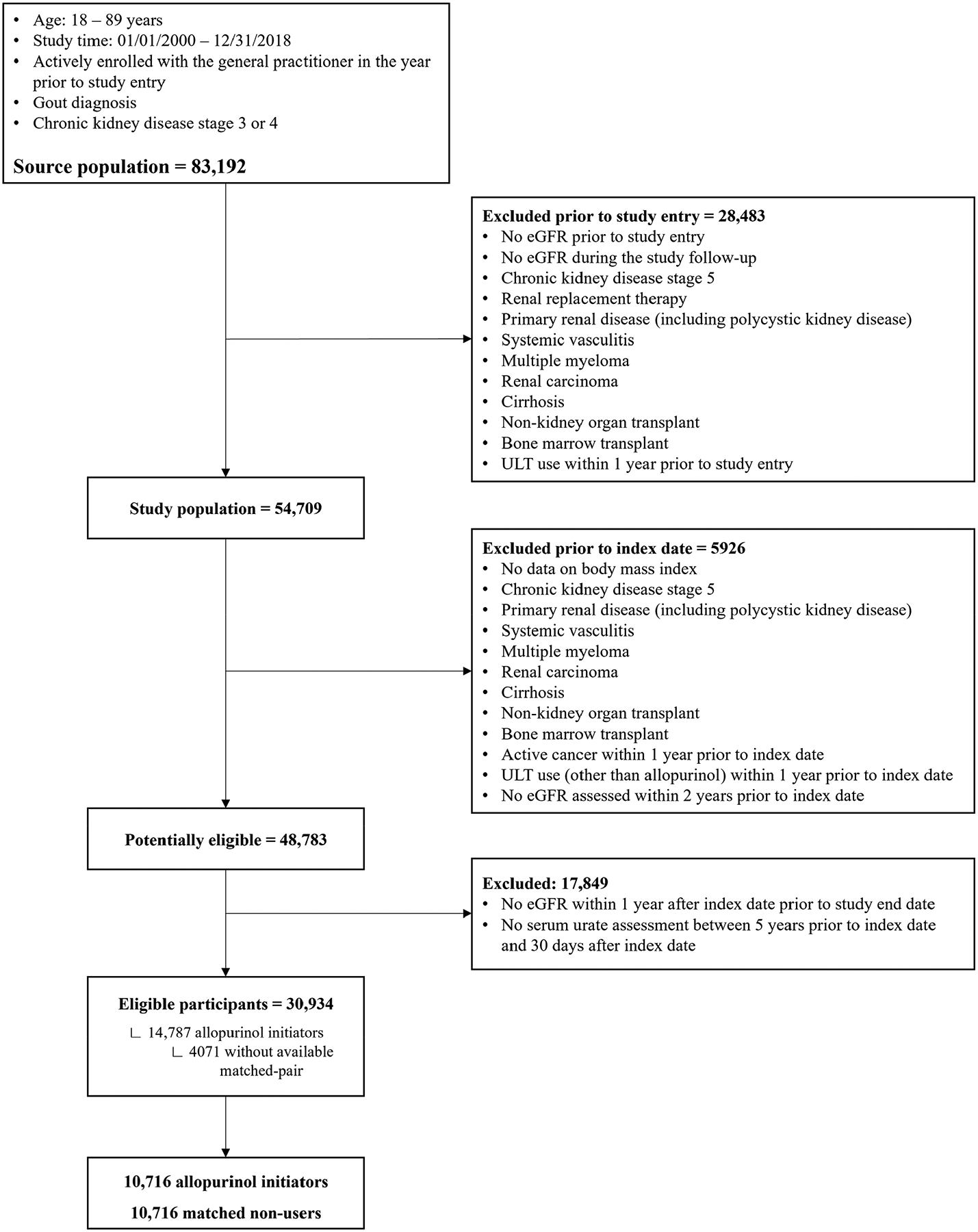
Flow diagram of the study participants. eGFR: estimated glomerular filtration rate; ULT: urate-lowering therapy.

The source population, i.e., participants who fulfilled all the five inclusion criteria, comprised 83,192 people (Figure 2). After applying all the exclusion criteria, 30,934 eligible participants remained. Among these individuals, there were 14,787 allopurinol initiators, with whom 10,716 non-allopurinol users could be matched, resulting in a study sample of 21,432 participants.

### 3.2. Participant Characteristics

About 58% were male, with a mean age of 74 years and a mean BMI of 30 kg/m^2^, with covariates well balanced between allopurinol initiators and non-initiators (Tables 1 and S2). Around 7% of each group were classified as CKD stage 4, whereas the remaining were classified as CKD stage 3. More than 90% of both groups had a diagnosis of cardiovascular disease, heart failure and/or hypertension. Almost half of the participants used NSAIDs at baseline.

### 3.3. Risk of CKD Progression Related to Allopurinol Use

The mean eGFR prior to the index date was 48.4 mL/min for allopurinol incident users and 49.5 mL/min for non-allopurinol users. At one year, the mean eGFR difference adjusted for the covariates in the propensity score model was 0.81 mL/min (95% CI 0.57–1.05), with a *p*-value < 0.0001 (Table 2), meaning that allopurinol initiators experienced a slightly greater improvement in the eGFR over the one-year follow-up period compared to the non-allopurinol users. While the absolute eGFR values remained similar between the two groups at the end of the follow-up, the allopurinol initiators showed a statistically significant, albeit small, improvement relative to the non-users.

### 3.4. Additional Analyses

Among the subjects initiating allopurinol therapy, 82% were prescribed an initial dose of 100 mg/day, while the remaining 18% received 300 mg/day; during the follow-up period, 36% of them had no SU measurements, and another 36% had only one. Ten allopurinol incident users (0.1%) and seven non-allopurinol users (0.1%) progressed to dialysis or renal transplant, while 381 (3.6%) and 399 (3.7%) respectively died within one year after the index date. During the follow-up time, incident allopurinol users had more visits to the GP and were also more exposed to colchicine (34.8% vs. 15.5%) and NSAIDs (35% vs. 26.1%) (Table S3).

The sensitivity analysis censoring the participants at exposure status change corroborated the primary findings, with a higher mean difference between incident allopurinol users and non-allopurinol users: 0.98 mL/min (95% CI 0.73–1.22, *p*-value < 0.0001).

## 4. Discussion

The community-based propensity score-matched cohort study of patients with gout and CKD stage 3–4 showed no detrimental effect of allopurinol initiation on renal function over a one-year period; in fact, a small beneficial effect of allopurinol use was noted, albeit of unclear clinical significance. Nonetheless, given the burden of gout among people with CKD and the fairly routine practice of ‘renal-dosing’ employed by many physicians yielding even poorer gout outcomes, the finding is important to highlight that no detrimental effect on renal function was noted.

This was in line with previous studies evaluating the effect of ULT on renal function [16–25]. The mechanisms through which allopurinol may be renoprotective include a possible direct effect through the inhibition of xanthine oxidase and indirect effects, such as controlling hyperuricemia and sparing NSAID use [2]. Unfortunately, the real-world data used in our study illustrate important inconsistencies with the recommended treatment of gout. We noted a high number of patients with gout who also have CKD who are not on ULT. Further, even among those on treatment, most patients did not have their allopurinol dose optimization guided by the SU target. Most concerning was the observation of frequent use of NSAIDs despite the presence of CKD. It is possible that the frequent use of NSAIDs could be partly attenuating the benefits of allopurinol treatment since gout flares may become more frequent in the first months of ULT.

Methodological issues have already been identified for the persistence of the renoprotective potential of ULT as an open question, including: many studies of ULT in hyperuricemia/gout exclude those with CKD; some investigations of ULT in CKD study those with gout and asymptomatic hyperuricemia as a mixed study sample; observational studies of gout are frequently unable to measure and balance gout severity between exposure arms; clinical trials of asymptomatic hyperuricemia frequently mix different CKD etiologic conditions that may not be wholly relevant to gout; some clinical trials of ULT and CKD include participants with normal levels of SU; many studies evaluate a specific fixed dose of ULT, regardless of the achieved SU, rather than a treat-to-target approach; most studies fail to provide the results stratified by kidney function; and many studies are limited in sample size or follow-up time.

Of note, two recent placebo-controlled randomized trials evaluated allopurinol in CKD [26,27]. The PERL study was conducted in those with type 1 DM, in which the relevance of hyperuricemia as the mechanism for renal disease is not clear [26]. The CKD-Fix study was conducted in patients with CKD stage 3 or 4 due to varied causes (most frequently diabetic kidney disease) who were considered to be at risk of progression, but with concerns about inadequate statistical power to detect differences [27]. Also important to note, both trials included participants with normal SU, excluded those with gout, and tested a fixed dose of allopurinol.

Our study also had some limitations. We were unable to ascertain medication adherence, which could reduce the ability to detect between-group differences if adherence is poor. Similarly, we are unable to comment upon whether lower levels of SU were associated with better renal function. The infrequent measurement of the eGFR in this cohort limits the identification of CKD progression over a short interval; however, we focused on a longer-term timeframe to minimize this concern. Gout severity could not be assessed, and we could expect greater disease severity among those who had allopurinol prescribed, potentially introducing a bias toward the null—disfavoring allopurinol in this analysis. Therefore, despite adjusting for a comprehensive set of potential confounders using propensity score matching, we acknowledge that unmeasured factors could contribute to residual confounding.

One strength of our study lies in the generalizability of our study sample, having been drawn from a data source that reflects the UK general population. The demographic characteristics of our sample reflect those of individuals with gout and CKD stage 3 or 4 encountered in general practice in the UK. Additionally, with our large sample size and methods to address confounding by indication and secular trends, these data provide some reassurance that allopurinol, at the very least, does not appear to be harmful to renal function among those with CKD stage 3 or 4.

## 5. Conclusions

In this community-based cohort, allopurinol use did not worsen renal function among patients with gout who had CKD 3–4 at baseline, and instead there was a suggestion of a small renoprotective effect. These findings support the use of allopurinol in the presence of CKD without concern for a detrimental renal effect.

## Supplementary Material

supplementary material

**Supplementary Materials:** The following supporting information can be downloaded at: https://www.mdpi.com/article/10.3390/gucdd3030013/s1, Table S1: Description of the covariates included in the propensity score; Methods Supplement; Table S2: Covariate balance; Table S3: Post-baseline characteristics of allopurinol initiators versus non-initiators; Table S4: Comparison of characteristics among excluded, eligible, and PS-matched participants.

## Figures and Tables

**Table 1.** Baseline characteristics of participants.

	Incident Allopurinol Users *n* = 10,716	Non-Allopurinol Users *n* = 10,716
*Demographics*		
Age, years, mean (SD)	74.1 (9.3)	74.2 (9.3)
Male, *n* (%)	6227 (58.1)	6187 (57.7)
Body mass index, kg/m^2^, mean (SD)	30.0 (5.6)	30.0 (5.8)
Hospitalization in year prior to index date, *n* (%)	2454 (22.9)	2450 (22.9)
Visits to the general practitioners in year prior to index date, *n* (%)		
0	396 (3.7)	409 (3.8)
1	425 (4.0)	420 (3.9)
2	666 (6.2)	663 (6.2)
3	803 (7.5)	794 (7.4)
4	877 (8.2)	912 (8.5)
5	905 (8.4)	911 (8.5)
6–7	1722 (16.1)	1729 (16.1)
8–10	1980 (18.5)	1994 (18.6)
≥11	2942 (27.5)	2884 (26.9)
*Comorbid conditions, n* (%)		
Baseline CKD stage 4	789 (7.4)	758 (7.1)
CVD/heart failure/hypertension	9834 (91.8)	9870 (92.1)
Diabetes	3091 (28.8)	3100 (28.9)
*Concomitant medication use, n (%)*		
Diuretics	7541 (70.4)	7661 (71.5)
ACEi/Non-losartan ARB	7654 (71.4)	7670 (71.6)
Losartan	714 (6.7)	688 (6.4)
Colchicine	3673 (34.3)	3685 (34.4)
Non-steroidal anti-inflammatory drugs	5232 (48.8)	5402 (50.4)
Aspirin	4671 (43.6)	4687 (43.7)
*Laboratory data, mean (SD)*		
Serum urate level, mg/dL	8.9 (1.6)	8.8 (1.6)

All these variables were used as covariates in the propensity score. ACEi: angiotensin-converting-enzyme inhibitor; ARB: angiotensin II receptor blockers; CKD: chronic kidney disease; CVD: cardiovascular disease; SD: standard deviation.

**Table 2.** Mean difference between groups in the eGFR change from baseline at one-year follow-up.

Main Results	Incident Allopurinol Users (*n* = 10,716)	Non-Allopurinol Users (*n* = 10,716)
Mean eGFR prior to index date (mL/min)	48.4	49.5
Mean eGFR within one-year post-index date (mL/min)	49.4	49.7
Mean eGFR change from baseline (mL/min)	1.0	0.2
Propensity score-matched mean difference between groups (95% CI)	0.82 (0.58–1.05), *p* < 0.0001	
**Additionally adjusted** * **mean difference between groups (95% CI)**	**0.81 (0.57–1.05), *p* < 0.0001**	

* Variables included in the propensity score model and included in the adjusted propensity score model: (1) general (age, gender, body mass index); (2) comorbidities (baseline CKD stage 4, cardiovascular disease/heart failure/hypertension, diabetes mellitus); (3) medication use (diuretics, angiotensin-converting-enzyme inhibitor/non-losartan angiotensin II receptor blockers, losartan, colchicine, non-steroidal anti-inflammatory drugs, aspirin); (4) visits to the general practitioner; (5) hospitalization; (6) serum urate. CI: confidence interval; CKD: chronic kidney disease; eGFR: estimated glomerular filtration rate.

## Data Availability

The original contributions presented in this study are included in the article/Supplementary Materials. Further inquiries can be directed to the corresponding authors.
