# Supplementary material for: Effect of Allopurinol Use on Kidney Function Among Patients with Gout and Chronic Kidney Disease
Source: Gout Urate Cryst Depos Dis. Author manuscript; Available in PMC 2025 Aug 23. (PMC12366798; doi:10.3390/gucdd3030013)
Supplement: supplementary material [file NIHMS2096728-supplement-supplementary_material.pdf]

Table S1. Description of the covariates included in the propensity score.

Covariates	Type	Ascertainment and Assessment Period
Age	continuous	At the index date
Gender	binary	Male / Female
BMI	continuous	Most recent BMI prior to the index date
Hospitalizations	binary	Yes / No<br>Within 1 year prior to the index date.
Number of visits to the general practitioner	categorical	Categories: 0, 1, 2, 3, 4, 5, 6-7, 8-10, 11+.<br>Within 1 year prior to the index date.
Diabetes	binary	Read codes or Prescription<br>• Read codes: any time prior to the index date.<br>• Prescription: within 1 year prior to the index date.
Chronic kidney disease stage 4	binary	Read codes or eGFR values<br>• Read codes: any time prior to the index date.<br>• eGFR value: most recent prior to or on the index date.
CVD / Heart Failure / Hypertension	binary	Read codes: any time prior to index date.
Medications:<br>• ACEi / Non-losartan ARBs<br>• Aspirin<br>• Colchicine<br>• Diuretics<br>• Losartan<br>• NSAIDs	binary	Prescription: within 1 year prior to the index date
Serum urate	continuous	The most recent value within 5 years prior to the index date. If there was no serum urate value within 5 years prior, then we used the first serum urate value within 30 days after the index date.

ACEi: angiotensin-converting-enzyme inhibitor; ARB: angiotensin II receptor blocker; BMI: body mass index; CVD: cardiovascular disease; eGFR: estimated glomerular filtration rate; NSAIDs: non-steroidal anti-inflammatory drugs.

Methods Supplement

Covariate balance in the PS-matched dataset was assessed by evaluating the standardized mean difference (SMD) using the SAS macro using %PMDIAG. The closer the SMD is to 0, the better is the covariate balance. Prior to PS-matching, 11 variables had SMD ≥ 0.1 (Table S2, in bold), ranging from 0.01 to 0.87.

In the PS-matched dataset, all SMD were lower than 0.1, ranging from <0.01 to 0.03, confirming a very good balance overall.

Table S2. Covariate balance.

**Covariates**	**SMD prior to PS-Matching**	**SMD after PS-Matching**
Age	**0.16**	0.01
Male	0.03	0.01
Body mass index	**0.24**	0.00
Baseline chronic kidney disease stage 4	**0.17**	0.01
Cardiovascular disease / heart failure / hypertension	0.05	0.01
Diabetes	0.01	0.00
Diuretics	**0.48**	0.02
ACEi / non-losartan ARBs	**0.17**	0.00
Losartan	0.05	0.01
Colchicine	**0.57**	0.00
Non-steroidal anti-inflammatory drugs	**0.30**	0.03
Aspirin	0.03	0.00
Number of visits to the general practitioner in prior year		
0	0.09	0.01
1	**0.11**	0.00
2	**0.10**	0.00
3	0.07	0.00
4	0.04	0.01
5	0.03	0.00
6-7	0.02	0.00
8-10	0.08	0.00
≥11	**0.16**	0.01
Hospitalization in prior year	0.08	0.00
Serum urate	**0.87**	0.02

ACEi: angiotensin-converting-enzyme inhibitor; ARB: angiotensin II receptor blockers; SMD: standardized mean difference.

Table S3. Post-baseline characteristics of allopurinol initiators versus non-initiators.

Visits to the general practitioner, number of serum urate measurements, colchicine use, non-steroidal anti-inflammatory drugs use, and allopurinol hypersensitivity syndrome were assessed between the index date and the post-index estimated glomerular filtration rate, whereas dialysis/kidney transplant and deaths were assessed within one year after the index date.

**Post-baseline characteristics**	**Incident allopurinol users N = 10,716**	**Non-allopurinol users N = 10,716**
Visits to the general practitioners, n (%)		
0	637 (5.9)	1,269 (11.8)
1	985 (9.2)	1,421 (13.3)
2	1,261 (11.8)	1,247 (11.6)
3	1,115 (10.4)	1,187 (11.1)
4	1,039 (9.7)	918 (8.6)
5	872 (8.1)	813 (7.6)
6-7	1,496 (14.0)	1,267 (11.8)
8-10	1,433 (13.4)	1,093 (10.2)
≥11	1,878 (17.5)	1,501 (14.0)
Number of serum urate measurements		
Mean (SD)	1.1 (1.3)	0.3 (0.7)
Median (IQR)	1 (0-2)	0 (0-0)
Range	0-15	0-8
Categorical, n (%)		
0	3,831 (35.8)	8,263 (77.1)
1	3,809 (35.5)	1,740 (16.2)
2	1,843 (17.2)	465 (4.3)
3	732 (6.8)	156 (1.5)
4	271 (2.5)	51 (0.5)
≥5	230 (2.1)	41 (0.4)
Colchicine	3,732 (34.8)	1,665 (15.5)
Non-steroidal anti-inflammatory drugs	3,750 (35.0)	2,792 (26.1)
Deaths, n (%)	381 (3.6)	399 (3.7)
Dialysis / kidney transplant, n (%)	10 (0.1)	7 (0.1)
Severe drug reaction, n (%)	22 (0.2)	6 (0.1)

IQR: interquartile range; SD: standard deviation.

Table S4. Comparison of characteristics among excluded, eligible, and PS-matched participants.

Participants	Excluded N = 20,831	Eligible N = 30,934	Matched N = 21,432
Demographics			
Age, years, mean (SD)	75.8 (9.4)	74.2 (9.2)	74.2 (9.3)
Male, n (%)	12,596 (60.5)	18,017 (58.2)	12,414 (57.9)
Body mass index, kg/m^2, mean (SD)	29.0 (5.5)	29.6 (5.6)	30.0 (5.7)
Hospitalization in year prior to index date, n (%)	4,641 (22.3)	6,707 (21.7)	4,904 (22.9)
Visits to the GP in year prior to index date, n (%)			
0	1,270 (6.1)	1,279 (4.1)	805 (3.8)
1	1,222 (5.9)	1,429 (4.6)	845 (3.9)
2	1,642 (7.9)	2,139 (6.9)	1,329 (6.2)
3	1,825 (8.8)	2,452 (7.9)	1,597 (7.5)
4	1,803 (8.7)	2,624 (8.5)	1,789 (8.3)
5	1,816 (8.7)	2,692 (8.7)	1,816 (8.5)
6-7	3,115 (15.0)	4,889 (15.8)	3,451 (16.1)
8-10	3,243 (15.6)	5,421 (17.5)	3,974 (18.5)
≥11	4,895 (23.5)	8,009 (25.9)	5,826 (27.2)
Comorbid conditions, n (%)			
Baseline chronic kidney disease stage 4	1,459 (7.0)	1,968 (6.4)	1,547 (7.2)
CVD / heart failure / hypertension	18,215 (87.4)	28,074 (90.8)	19,704 (91.9)
Diabetes mellitus	5,555 (26.7)	8,631 (27.9)	6,191 (28.9)
Concomitant medication use, n (%)			
Diuretics (loop, thiazide, thiazide-like)	12,702 (61.0)	20,130 (65.1)	15,202 (70.9)
ACEi / non-losartan ARBs	12,633 (60.6)	21,010 (67.9)	15,324 (71.5)
Losartan	1,312 (6.3)	2,086 (6.7)	1,402 (6.5)
Colchicine	3,564 (17.1)	8,590 (27.8)	7,358 (34.3)
Non-steroidal anti-inflammatory drugs	7,919 (38.0)	14,207 (45.9)	10,634 (49.6)
Low dose aspirin	9,141 (43.9)	13,255 (42.8)	9,358 (43.7)
Laboratory data			
Serum urate level, mg/dL, mean (SD)	8.4 (1.9)	8.5 (1.8)	8.8 (1.6)

ACEi: angiotensin-converting-enzyme inhibitor; ARB: angiotensin II receptor blockers; CVD: cardiovascular disease; GP: general practitioner; PS: propensity-score; SD: Standard deviation.
